# PCNA dependent cellular activities tolerate dramatic perturbations in PCNA client interactions

**DOI:** 10.1016/j.dnarep.2016.12.003

**Published:** 2017-02

**Authors:** Rosemary H.C. Wilson, Antonio J. Biasutto, Lihao Wang, Roman Fischer, Emma L. Baple, Andrew H. Crosby, Erika J. Mancini, Catherine M. Green

**Affiliations:** a Wellcome Trust Centre for Human Genetics, University of Oxford, Roosevelt Drive, Oxford, OX3 7BN, UK; b Division of Structural Biology, Wellcome Trust Centre for Human Genetics, University of Oxford, Roosevelt Drive, Oxford, OX3 7BN, UK; c Target Discovery Institute, University of Oxford, Roosevelt Drive, Oxford OX3 7FZ, UK; d University of Exeter Medical School, Barrack Road, Exeter, EX2 5DW, UK; e School of Life Sciences, University of Sussex, Falmer, Brighton, BN1 9RH, UK

**Keywords:** PCNA, PCNA-associated repair disorder (PARD), DNA replication, DNA repair

## Abstract

Proliferating cell nuclear antigen (PCNA) is an essential cofactor for DNA replication and repair, recruiting multiple proteins to their sites of action. We examined the effects of the PCNA^S228I^ mutation that causes PCNA-associated DNA repair disorder (PARD). Cells from individuals affected by PARD are sensitive to the PCNA inhibitors T3 and T2AA, showing that the S228I mutation has consequences for undamaged cells. Analysis of the binding between PCNA and PCNA-interacting proteins (PIPs) shows that the S228I change dramatically impairs the majority of these interactions, including that of Cdt1, DNMT1, PolD3^p66^ and PolD4^p12^. In contrast p21 largely retains the ability to bind PCNA^S228I^. This property is conferred by the p21 PIP box sequence itself, which is both necessary and sufficient for PCNA^S228I^ binding. Ubiquitination of PCNA is unaffected by the S228I change, which indirectly alters the structure of the inter-domain connecting loop. Despite the dramatic in vitro effects of the PARD mutation on PIP-degron binding, there are only minor alterations to the stability of p21 and Cdt1 in cells from affected individuals. Overall our data suggests that reduced affinity of PCNA^S228I^ for specific clients causes subtle cellular defects in undamaged cells which likely contribute to the etiology of PARD.

## Highlights

• We assess the cellular effects of the mutation that causes PARD (PCNA^S228I^).
• Cells from affected individuals are sensitive to T2AA and T3.
• PCNA^S228I^ impairs interactions between PCNA and Cdt1, DNMT1, PolD3 and PolD4.
• The PIP-box of p21 retains binding to PCNA^S228I^.
• PCNA-dependent degradation and the cell cycle are only subtly altered by PCNA^S228I^.

## 1 Introduction

Accurate DNA replication is essential prior to cell division if daughter cells are to inherit a genome free from mutation. The proliferating cell nuclear antigen (PCNA) has a central role during DNA replication, acting to recruit enzymes to the DNA replication fork, and to modify enzymatic activity and processivity. At replication sites PCNA recruits proteins required throughout the complex processes of chromosomal duplication, including (but not limited to): DNA polymerase delta (for lagging strand synthesis) [1], DNA ligase 1 (Lig1) and Flap endonuclease 1 (Fen1) (for Okazaki fragment maturation) [2], [3], [4], RNaseH2B (for ribonucleotide removal) [5], [6], [7], polymerase eta (Polη) (for translesion synthesis) [8], DNA methyltransferase 1 (DNMT1) (for DNA methylation maintenance) [9], and chromatin assembly factor 1 (for chromatin assembly) [10], [11]. PCNA also regulates entry into S-phase via interactions with the cell cycle regulator p21 [12] and the pre-replication complex component Cdt1 [13]. These PCNA partners all utilise a similar peptide motif to associate with PCNA, the PCNA-interacting-protein (PIP) box. This short motif interacts via a combination of charge-pair and hydrophobic interactions with the interdomain connecting loop (IDCL) of PCNA [14], [15]. Proteins that bind PCNA in this manner therefore compete for the same interaction surface, perhaps to enable dynamic associations essential for progressing through the multiple stages of chromosomal replication [16]. It has often been postulated that PCNA might coordinate DNA replication, the trimeric nature of PCNA lending itself to a “toolbelt model” where, by binding multiple proteins sequentially, PCNA ensures appropriate selection of enzymes at the replication fork [17]. However, this model has recently been challenged by work showing that PCNA with only a single interaction surface is still functional [18].

As well as its essential role in DNA replication, PCNA is also required for DNA repair. PCNA is involved in the processes of nucleotide excision repair (NER), long-patch base excision repair (BER) and mismatch repair (MMR) [16]. During these processes PCNA interacts directly with repair proteins, including (but not limited to): xeroderma pigmentosum A and G (XPA, XPG) (for NER) [19], [20], DNA glycosylases (UNG2 and MPG), endonucleases (APE1 and APE2) and polymerase beta [21], [22], [23], [24], [25] (for BER), and MutS homologs 3 and 6 (for MMR) [26]. It is also possible that PCNA is required for DNA repair synthesis during homologous recombination [27].

As PCNA is a crucial component of important pathways for DNA metabolism, it is not surprising that the encoding gene is essential in yeast and mice [28], [29], [30]. The gene is highly conserved from yeast to humans; *S. cerevisiae*, *S. pombe* and *M. musculus* PCNAs are 35%, 51% and 97% identical to the human protein, respectively [EMBOSS Needle]. Site-specific mutations of the *S. cerevisiae* protein result in a variety of phenotypes, including cold sensitivity, sensitivity to DNA damaging agents and alterations to telomere position effects [31], [32], [33], [34]. In mice the only characterised PCNA variant is the site directed mutation of lysine-164 to arginine, which results in infertility and in alterations to the somatic hypermutation spectrum due to the requirement for ubiquitination on PCNA Lys164 for the recruitment of Polη [35]. The PCNA protein is not invariant in the human population, but its variation is very low. There are only seven missense coding SNPs reported in the 1000 genomes browser (rs140522967, Ser223Pro; rs369958038, Ser228Ile; rs376351202, Met139Val; rs141842220, Ala67Thr; rs144468297, Asn65Thr; rs1050525, Ser39Arg; and rs375496467, Val15Leu) all with minor allele frequencies of less than 0.01 (where reported). Of these only one very rare allele (rs369958038, S228I) is reported pathogenic in the homozygous state [36]. We previously described four individuals from the Ohio Amish population who are homozygous for this S228I sequence alteration and affected by PCNA-associated DNA repair disorder (PARD), characterised by short stature, hearing loss, premature aging, telangiectasia, neurodegeneration and photosensitivity [36]. A further PARD affected Amish individual from Wisconsin homozygous for the same S228I founder mutation has since been identified, she presented aged 4 years with short stature, sun sensitivity, progressive gait instability and hearing concerns. On examination, there was no evidence of ocular or cutaneous telangiectasia, which appear to be a later manifestation of the disease.

We previously showed that PCNA^S228I^ protein has altered binding to a number of client proteins, in particular XPG, Lig1 and Fen1, and that cells from PARD affected individuals were more sensitive to UV damage [36]. We here show data that PCNA^S228I^ also causes repair independent consequences in cells from PARD affected inidividuals and present in-depth characterisation of the protein binding capability of PCNA^S228I^, showing that the effect on binding varies significantly across a range of PCNA interactors, dependent on the sequence of the PIP-box. These consequences for cellular functions will shed light on the complex pathology of this disorder.

## 2 Material and methods

### 2.1 Cell lines

EBV transformed lymphoblastoid cell lines were established from four PARD affected individuals (1504, 1505, 1506, 1779) and two Amish wild type controls (0920, 0924) using the service from Public Health England. Cell lines were maintained in RPMI with 10% FBS, 2 mM glutamine (Sigma), and 1% penicillin and streptomycin (PAA). Sensitivity of lymphoblasts to T2AA [37] (T2 amino alcohol ((*S*)-4-(4-(2-amino-3-hydroxylpropyl)-2,6-diiodophenoxyphenol)) and T3 (3,3′,5-triiodothyronine) was determined by addition of T2AA or T3 to 5 ml cells at 2 × 10^5^/ml and counting viable using trypan blue exclusion at indicated time points.

### 2.2 Genotyping

Genotypes were confirmed regularly for quality control purposes. Genomic DNA was extracted from lymphoblastoid cell lines using GenElute Mammalian Genomic DNA Miniprep Kit as per manufacturer’s instructions. The genomic region surrounding PCNA nucleotide position 683 was amplified using standard PCR with Phusion^®^ High Fidelity DNA Polymerase (NEB, as per manufacturer’s instructions), using the following primers 5′-ATAGCTCCCTCCAAAGTGACC-3′ and 5′-CATCCTCGATCTTGGGAGCC-3′. Amplification of a single band was confirmed by agarose gel electrophoresis and samples sequenced by Sanger sequencing (GATC Biotech) using the primer 5′-ACTAACTTTTGCACTGAGG-3′.

### 2.3 FACS analysis of cell cycle

Exponentially growing lymphoblastoid cells were incubated with 10 μM Bromodeoxy-uridine (BrdU) for 30 min then centrifuged (1000*g*, 5 min) and fixed in 4% paraformaldehyde (PFA), 20 min. Cells were washed in PBS, treated with 0.2 mg/mL pepsin in 2 M HCl, 20 mins, then probed with anti-BrdU (1:50, 347580, BD) 1 h in PBS with 1% BSA and 0.5% Tween 20 with intervening washes with PBS. Cells were washed in PBS with 1% BSA, 0.5% Tween 20 and incubated with Alexa Fluor 633 anti-mouse (1:200, Invitrogen) for 1 h. Cells were washed as before, incubated in PBS with 0.5 mg/mL RNase A overnight at 4 °C and stained with 10 μg/mL propidium iodide before analysis using a CyAn ADP Analyzer (Beckman Coulter).

### 2.4 Cell manipulations

For analysis of PIP degrons, exponentially growing lymphoblasts were treated with indicated levels of 254 nm UVC light in PBS. Cells were allowed to recover in growth medium for the indicated times, then harvested at 4 °C for protein analysis. Samples were prepared in Benzonase buffer (25 mM NaCl, 50 mM HEPES pH 7.8, 0.05% SDS, 4 mM MgCl_2_, 5x cOmplete EDTA free Protease Inhibitor Cocktail Tablets (Roche), 0.5% Benzonase (Novagen)) with incubation on ice for 10 mins. Protein levels were adjusted by dilution using Bradford Reagent and 4x Laemmli added, 95 °C, 5 mins. Proteins were separated on SDS-PAGE gels and transferred to 0.2 μm nitrocellulose.

### 2.5 Antibodies

The following primary antibodies were used for western blotting mouse anti-actin (A4700, Sigma Aldrich), PCNA (PC10, Millipore), rabbit anti-Cdt1 (D10F11, Cell Signalling Technology), DNMT1 (NB100-56519, Novus Biologicals), Fen1 (EPR4459(2), GeneTex), Ligase I (PA5-27820, Thermo Scientific), p21 (EPR362, abcam), Polη (ab17725, abcam), Ubiquitinated-PCNA (D5C7P, Cell Signalling Technology).

### 2.6 Mass spectrometry

HEK293 stably expressing StrepTagII V5 PCNA^WT^ or empty vector control were made using pEXP pcDNA3.1/StrepTagII V5 PCNA^WT^ generated from pcDNA3.1/nV5-DEST (ThermoFisher) with StrepTagII inserted at HinDIII site and PCNA inserted by LR reaction (ThermoFisher). Stable clones were selected with Neomycin and selected for level of exogenous PCNA expression by western blot. Cells were cultured and lysates collected in extract buffer (0.5% Igepal, 40 mM NaCl, 50 mM Tris pH 7.5, 2 mM MgCl_2_, 2x cOmplete Protease Inhibitor Cocktail Tablets (Roche), 1x phosphatase inhibitor (Roche), 0.1% Benzonase (Novagen)) on ice, 10 min. NaCl was increased to 150 mM with 20 min incubation, 4 °C and lysates cleared 21000 g, 4 °C, 20 min. Protein concentrations were standardised using Bradford assay then incubated with Strep-Tactin Superflow Plus (Qiagen) for 1 h at 4 °C in binding buffer (0.5% Igepal, 150 mM NaCl, 50 mM Tris pH 7.5, 2 mM EDTA, 1x cOmplete Protease Inhibitor Cocktail Tablet (Roche)). Beads were washed in binding buffer and eluted with NuPAGE LDS sample buffer and NuPAGE reducing agent (both Thermo). Start and unbound samples were also prepared. Bound proteins were separated using NuPAGE Midi 4–12% SDS-PAGE gel (Thermo), stained with Safe Stain (Invitrogen), bands corresponding to endogenous and Strep-V5-PCNA were excised and analysed by Mass Spectrometry as described previously [38]. Briefly, gelbands were destained with 50% methanol in 5% acetic acid before reduction (10 mM DTT), alkylation (50 mM iodoacetamide) and digest with Trypsin. Extracted peptides were analysed with a nAcquity (Waters) coupled Orbitrap Elite (Thermo) LC–MS/MS system. The peptide mixture was separated using a gradient of 2–40% Acetonitrile in 0.1% formic acid. Precursors between 300 and 1500 *m*/*z* were isolated with a mass window of 1.8 *m*/*z* and analysed after CID fragmentation in the ion trap with a normalized collision energy of 35%. RAW data was analysed with PEAKS (Bioinformatics Solutions) version 7 using 10 ppm precursor and 0.5 Da fragment mass accuracy. We allowed to search for phosphorylation (S, T, Y), deamidation (N and Q) and oxidation (M) as variable modifications and carbamidomethylation (C) as fixed modification. Peptide FDR was set to 1%.

### 2.7 Recombinant protein production and purification

Recombinant His-S-tagged PCNA^WT^ and PCNA^S228I^ were produced using pET30a, recombinant GST-PIP box fusions using pGEX4T-1 (GE Healthcare) and ‘3tag’ PCNA^WT^ and PCNA^S228I^ were produced using co-transfection of pEXP GST PCNA^WT^ or PCNA^S228I^ (pGEX6P-1, GE Healthcare), pRSFDuet-1 (Novagen) with the S-tag exchanged for the AviTag™ (Avidity) sequence, expressing either His-PCNA^WT^ and AviTag™-PCNA^WT^ or His-PCNA^S228I^ and AviTag™-PCNA^S228I^, and pBirA (Avidity) to biotinylate the AviTag™ during production. All plasmids were verified by sequencing. Proteins were expressed at OD600 ∼ 0.6-0.8 with 0.1 mM IPTG at 25 °C for 5 h in E.coli BL21 codonplus (Novagen).

His-S-tagged PCNA was purified using Ni-NTA sepharose (QIAGEN) and GST-PIP box fusions were purified using Glutathione Sepharose 4B (GE Healthcare). ‘3tag’ PCNA was purified sequentially using Ni-NTA sepharose, Glutathione sepharose and eluted from the column using PreScission Protease (GE Healthcare). All proteins were further purified by Size Exclusion Chromatography using HiLoad 16/60 Superdex 200 or 75 (GE Healthcare) in HBS-EP buffer as appropriate for Surface Plasmon Resonance.

Untagged PCNA^S228I^ was generated using site directed mutagenesis (QuikChange XL, Agilent Technologies) using previously published untagged PCNA vector [7], confirmed by sequencing. Untagged PCNA^S228I^ was expressed in E.coli BL21 Star (DE3) (ThermoFisher) using 1 mM IPTG at 18 °C for 16 h.

Protein purification of untagged PCNA^S228I^ for crystallography followed Ludwig et al., 2010 [39] with modifications. For all steps, fractions containing the protein were identified via SDS-PAGE analysis. Pellets were harvested and resuspended to 0.1 mg/mL in 20 mM Tris pH 8.0 and 50 mM NaCl, supplemented with cOmplete EDTA-free Protease Inhibitor Cocktail (Roche), 80 U/mL DNase I (Sigma) and 0.25 mg/mL Lysozyme (Sigma). Cells were lysed via cell disruption and cleared through centrifugation at 4 °C and 48000*g* for 1 h, the supernatant was filtered and loaded on a pre-equilibrated 5 ml Ion Exchange Q FF column (GE Healthcare) using an ÄKTA Purifier system (GE Healthcare). The column was washed and all bound proteins were eluted over a complex, 4-step gradient profile: 5CV linear gradient from 50 mM NaCl (0% B) to 300 mM NaCl (27% B), followed by a 3CV gradient to 560 mM NaCl (54% B), then a 5CV linear gradient to 590 mM NaCl (57% B), finishing with a 5CV linear gradient to 1 M NaCl (100% B). Fractions containing the protein were pooled and buffer exchanged to 30 mM NaOAc pH 5.0 and 5 mM NaCl using a HiLoad 10/60 Desalting column. The protein mixture was then flowed through a pre-equilibrated 5 ml Ion Exchange Heparin column (GE Healthcare), to which PCNA^S228I^ did not bind. The Heparin flow-through was buffer exchanged to 20 mM Tris pH 8.0 and 50 mM NaCl using a HiLoad 10/60 Desalting column, refocused by binding to a pre-equilibrated 5 ml Ion Exchange Q FF column and eluted over 10CV a linear gradient to 1 M NaCl. Protein eluate fractions were pooled and further purified by Size Exclusion Chromatography, using a HiLoad 16/600 Superdex 200 column (GE Healthcare) equilibrated in 25 mM Tris pH 8.0, 25 mM NaCl, 0.5 mM EDTA, 10% Glycerol. Fractions containing the protein were pooled and concentrated to ∼6.65 mg/mL (monomer) for crystallisation using an Amicon 50 kD MWCO filter (Millipore).

### 2.8 GST pull downs

GST pulldown experiments were performed as previously described [19] except recombinant proteins were purified prior to the experiment. 10 μg (or 100 μg for weak binders as indicated) GST-PIP fusions, 20 μg His-S-PCNA or His-S-PCNA^S228I^ and 62.5 μg BSA were combined in a buffer containing 100 mM KH_2_PO_4_/K_2_HPO_4_. 10% was removed as ‘start’, the rest incubated with Glutathione sepharose 4B beads (GE Healthcare) for 2 h at 4 °C. Beads were washed thoroughly, then boiled in Laemmli buffer. 10% of input samples and 25% of bead samples were separated by SDS-PAGE and stained with Coomassie. For western blotting, samples were diluted 1:20, separated by SDS-PAGE, transferred to 0.2 μm nitrocellulose and probed for PCNA.

### 2.9 Surface plasmon resonance (SPR)

SPR was performed in two orientations using a Biacore T200 machine (GE Healthcare). For data in Fig. 3 and Supplementary Fig. 2 ∼1000 Response Units (RU) GST-PIP fusions (ligand) were bound to each lane of a CM5 S series chip (GE Healthcare) in acetate pH 5.5 buffer using the Biacore immobilisation wizard with GST as a control lane. Binding of the analytes, His-S-PCNA constructs, was investigated using equilibrium of binding method in HBS-EP buffer (0.01 M HEPES pH 7.4, 0.15 M NaCl, 3 mM EDTA, 0.0005% IGEPAL) with 1 g/l BSA, filtered and de-gassed. The method used 240 s contact time followed by 600 s dissociation with low sample consumption setting and flow rate of 20 μl/min. A 10 point 2x dilution series from 12 μM was used including at least one 0 μM sample, from low to high to low, repeating each concentration except 12 μM. Affinity curves were plotted by subtracting RU from the GST lane from the lane of interest and subtracting blank analyte conditions, using Biacore T200 Evaluation software using surface bound affinity and steady state affinity fit options. Data was collected at 10 Hz and equilibrium of binding was taken in a 5 s window, 4 s before the injection stopped.

For reverse SPR (Supplementary Fig. 3), ∼1000 RU 3tag-PCNA^WT^ or PCNA^S228I^ were immobilised on an SA S series chip (GE Healthcare) using SA immobilisation wizard. Binding of GST-PIP fusions (analyte) was investigated using a Single Cycle Kinetics method in HBS-EP with 1 g/l BSA, filtered and de-gassed. The SCK method consisted of 5 binding events of increasing concentration from a 2x dilution series with one internal repeat (Cdt1, highest concentration 44.5 μM) or 10 binding events in two 5 step cycles with one internal repeat (p21 highest concentration 27.9 μM, GST highest concentration 53.2 μM). After each 5 step cycle was run, the same cycle was repeated with buffer only. Contact time was 250 s, dissociation time of 600 s, at 20 μl/min with an additional 10 s buffer contact and 1800s, 30 μl/min dissociation after each 5 step cycle. GST and p21 cycles were run with an additional attempted ‘regeneration step’ of 30 s 100% ethylene glycol 30 μl/min, high viscosity, 60 s stabilization. However, we could not find suitable regeneration conditions so data was only collected from fresh chips.

### 2.10 Crystallisation, data collection and structure determination

Sitting drop, vapour diffusion crystallisation experiments were set up in 96-well plates (Greiner) using a nanoliter Robot (Cartesian Technologies) [40]. Diffraction quality crystals in P4_3_2_1_2 space group grew after 10 days at 20 °C, set up in a 1:1 protein:precipitant volume ratio with well buffer containing 2 M (NH_4_)_2_SO_4_ and 0.1 M NaOAc pH 4.5. Crystals were cryo-protected using 20% (^v^/_v_) Ethylene glycol in crystallisation buffer, before flash-cooling in liquid nitrogen. Data up to 2.27 Å was collected on the i03 beamline at the Diamond Light Source, Didcot, UK. Data was processed and scaled using Xia2 [41] and the structure was solved by molecular replacement using PHASER [42] with the APO structure of wild-type PCNA as a search model (1VYM [43]), iterative rounds of manual building with Coot [44] and refinement using Phenix [45] with TLS parameters, Secondary Structure and Non-Crystallographic Symmetry restraints. Structures were validated with MolProbity [46] resulting in an overall score of 1.31 at 2.27 Å. Data collection and refinement statistics are detailed in Table 1, Protein Database Reference 5MOM.

## 3 Results

### 3.1 Cells from PARD affected individuals are more sensitive to the PCNA-interaction competitors T3 and T2AA

Many of the proteins that we identified as having altered interactions with PCNA^S228I^ are involved in both DNA repair and DNA replication. While we found significant repair deficiencies in cells carrying the PARD mutation we did not identify any replication related defects. Therefore the weakened interactions that occur in the PCNA^S228I^ background must still be sufficient to allow the essential activity of PCNA under unperturbed conditions. However, this generally reduced ability of PCNA to bind PIP box containing proteins in PCNA^S228I^ cells might mean that PIP-PCNA interactions in this genetic background would be particularly sensitive to additional competition. Two small molecule inhibitors of PIP-PCNA interactions have been previously reported: T3 (3,3′,5-triiodothyronine) and T2AA (T2-aminoalcohol hydrochloride) [37]. Structural analysis showed that these two compounds interact with PCNA at the IDCL, blocking PIP binding. We therefore hypothesised that PCNA^S228I^ containing cells would be more sensitive to these inhibitors.

**Fig. 1:**
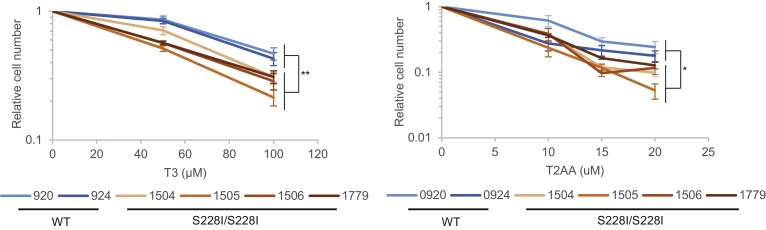
PCNA^S228I^ cells are sensitive to increased competition for IDCL binding. Graphs show relative cell number for PCNA^S228I/S228I^ and PCNA^WT/WT^ lymphoblasts treated with indicated concentrations of T3 (left) or T2AA (right) for three days. Data expressed as cell number relative to cells treated with vehicle. Data are average of at least three experiments, error bars are SEM, * p < 0.05, ** p < 0.005 using Student’s *t*-test.

We assessed the sensitivity of EBV-transformed lymphoblasts from four PARD-affected individuals (1504, 1505, 1506, 1779) and two unaffected controls (0920, 0924) to T3 and T2AA using cell proliferation assays, and found that all four PCNA^S228I^ cell lines had reduced growth in the presence of T3 and T2AA compared to WT lines (Fig. 1). The differences between the genotypes are statistically significant (p = 0.0091 at 100 μM T3 and p = 0.024 at 20 μM T2AA). This implies that PCNA^S228I^-PIP interactions are already limited in an undamaged context and that increasing competition for PCNA’s key binding site, the IDCL, is particularly toxic in this situation. We note that the concentrations of T3 (a thyroid hormone) used to inhibit PCNA-PIP interactions are at least a thousand fold higher than the blood concentration in normal adults (equivalent to 1.2–3 nM) making it unlikely that the symptoms of PARD are related to T3-dependent PCNA inhibition. However, these data do suggest that cells from affected individuals are likely to be sensitive to even transient and localised perturbations to PCNA client binding events. This is the first in vivo indication that some of the symptoms of PARD might derive from a reduced ability of PCNA^S228I^ to function in a repair independent manner and this may well contribute to the wide-ranging clinical manifestations of PARD. We thus investigated the effect of PCNA^S228I^ on a range of other PCNA interactors.

### 3.2 The affinities of PCNA-client interactions are reduced to varying extents by the PCNA^S228I^ change

**Fig. 2:**
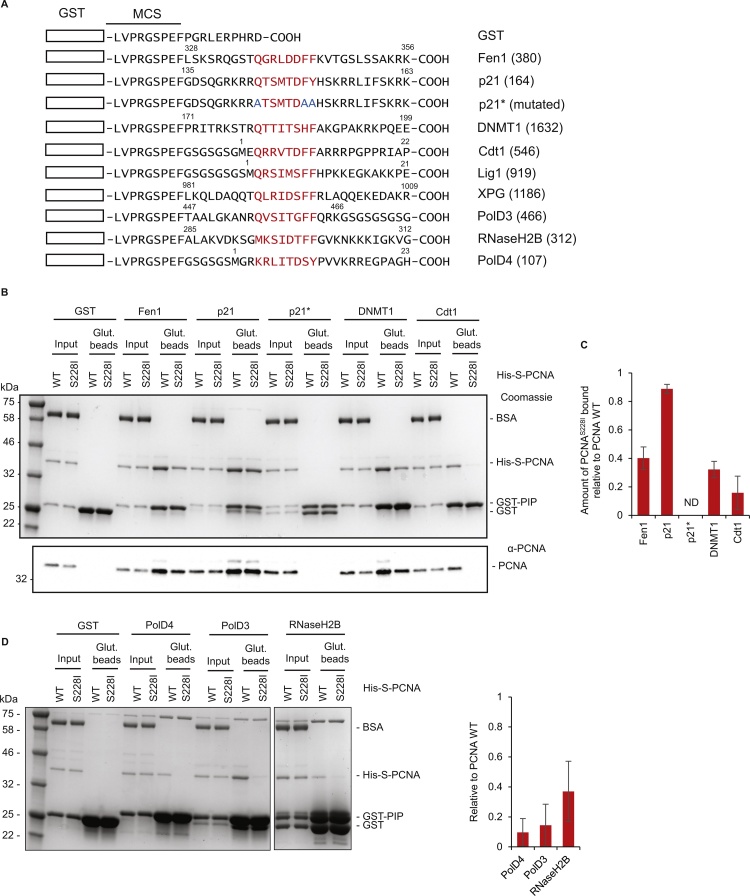
The PCNA^S228I^ mutation differentially affects PCNA binding to PIP box-containing proteins. A) Schematic showing GST-PIP peptide constructs generated during this study. GST represented by rectangle, PIP box sequences shown in red, mutated residues shown in blue. Numbers relate to amino acid position in full length protein. B) GST-PIP pull down of His-S-PCNA^WT^ or PCNA^S228I^. Figure shows Coomassie stained gel of representative pull down (top) and anti-PCNA western blot of the same samples diluted 1:20 (bottom). Amount of ‘input’ loaded for Coomassie is equivalent to 1%, ‘Glut. beads’ (Glutathione sepharose 4B beads) is equivalent to 25%. Molecular weight markers are indicated. C) Quantification of binding. Histogram shows amount of PCNA^S228I^ pulled down relative to PCNA^WT^ for indicated PIP constructs. Fen1 (n = 8), p21 (n = 8), p21* (n = 1), DNMT1 (n = 3), Cdt1 (n = 6). Error bars are standard deviation. D) GST-PIP pull down of His-S-PCNA^WT^ or PCNA^S228I^ for weak binders with 100 μg GST-PIP construct used per condition. Coomassie as B, quantification as C, n = 3.

To analyse the differences in the binding capabilities of PCNA and PCNA^S228I^ we used a GST-PIP box pull down assay. As we previously showed for Fen1, the PIP boxes of Cdt1 and DNMT1 are severely reduced in their ability to bind PCNA^S228I^ (Fig. 2). Given our previous SILAC analysis of PCNA^S228I^ interactions we were surprised that the DNMT1 binding was so dramatically affected, as endogenous DNMT1 bound equally to PCNA^WT^ and PCNA^S118I^ columns [36]. This may imply that additional binding sequences are present in the full length DNMT1 protein or simply differences in the experimental approaches used. We also observed reduced binding of RNaseH2B, PolD3^p66^ and PolD4^p12^ PIP boxes to PCNA^S228I^ although all are weak binders requiring 10-fold higher input of GST-PIP proteins to observe binding to PCNA WT (Fig. 2D). For RNaseH2B and PolD3^p66^ these data support our previous SILAC dataset [36], and in the case of RNaseH2B this also supports ITC data in a recent paper from Duffey et al. [47]. However, the associations between Cdt1, p21 or PolD4^p12^ and PCNA^S228I^ were not determined in our previous study, presumably due to their low abundance in undamaged asynchronous cell extracts.

In contrast to the other PIP boxes tested, the PCNA binding of the p21-PIP-box is only minimally affected by PCNA^S228I^ (Fig. 2B, C). This retained ability of p21 to bind PCNA^S228I^ was also observed using ITC by Duffey et al. [47]. As with the other clients tested here, the binding of the p21 PIP-box to both PCNA^WT^ and PCNA^S228I^ is mediated by the canonical PIP-IDCL interface as both interactions were abolished when the key PIP-box residues (corresponding to Glu144, Phe150, Tyr151 in the full length protein) were mutated to alanine (Fig. 2B). We also used the GST pulldown approach to test the binding of other PIP-boxes to PCNA^S228I^. However, we saw no binding of GST-PIP fusion proteins derived from Cdc6, or Cdt2 even to PCNA^WT^ (Supplementary Fig. 1A, B). These PIP boxes presumably comprise a sub-class with binding avidity below the sensitivity threshold of our assay, or requiring specific buffer conditions (although we also tested these under the published buffer conditions, Supplementary Fig. 1C) or additional binding motifs.

**Fig. 3:**
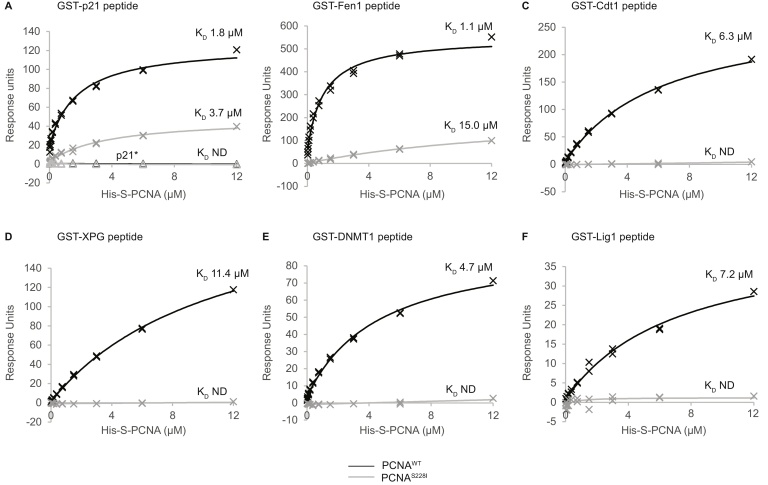
The affinity of PCNA-PIP box interactions is reduced by PCNA^S228I^ variant. Affinity curves generated from SPR data for His-S-PCNA^WT^ or His-S-PCNA^S228I^ binding to CM5 chip coupled to indicated GST-PIP peptide. PCNA^WT^ is shown in black, PCNA^S228I^ in grey. GST-mutated p21 PIP peptide (GST-p21*) is also shown on the p21 graph (A) with triangle marker points; no binding of either PCNA^WT^ or PCNA^S228I^ to GST-p21* peptide lane was observed. K_D_ calculated from Biacore T200 evaluation software indicated on relevant curves. ND = not determined.

To obtain a quantitative measure of the effect of the PCNA^S228I^ mutation on client protein binding we turned to surface plasmon resonance (SPR). GST-PIP-box fusion proteins from Cdt1, DNMT1, p21, XPG, Lig1, and Fen1 were coupled to a CM5 chip, and the binding of analyte PCNA (His-S-tagged, either WT or S228I) was assessed. PCNA^WT^ associated with all the GST-PIP-box fusion proteins, except that in which the interaction site had been mutated (p21*) (Fig. 3, Supplementary Fig. 2). In all cases the affinity of the PCNA for the PIP-box fusions was reduced by the PCNA^S228I^ change (Fig. 3), but the magnitude of these changes differed greatly; from ∼2 fold for the p21 PIP-box (K_D_ increased from 1.8 μM to 3.7 μM) to ∼14 fold for the Fen1 PIP-box (1.1 μM to 15 μM) (Fig. 3A, B). In fact, in most cases (Cdt1, XPG, DNMT1, Lig1) the binding of PCNA^S228I^ to the PIP-box fusions was too low for a K_D_ to be determined (Fig. 3C–F). Similar results were obtained when we coupled biotinylated '3-tag'-PCNA to a streptavidin coated chip surface and used GST fused to the PIP-box from p21 or Cdt1 as the analyte. We note that, in this orientation, the affinity of PCNA for the p21 PIP was only reduced 10% by the S228I change (Supplementary Fig. 3).

In many cases we are able to detect low levels of PCNA^S228I^ binding to PIP-box fusion proteins by GST pulldown even when the same interactions are not detectable by SPR. This is presumably due to biophysical differences in assay format. By pulldown, the interactions between PCNA^S228I^ and the PIP-boxes of Cdt1 and DNMT1, are low, but detectable above background (Fig. 2B, C and Supplementary Fig. 1D), like XPG, Lig1 and Fen1 [36]. However, it is clear that the striking effect of the PCNA^S228I^ change is apparent in both of these very different assays. We note that in Xenopus cell free extracts the binding of Cdt1 to PCNA has been suggested to be dependent on DNA [48], however other data shows interaction between recombinant Cdt1 and PCNA [13]. Here, the addition of DNA to GST pull down assays had no effect on the Cdt1 PIP box pull down of either PCNA^WT^ or PCNA^S228I^ (Supplementary Fig. 1E).

These data demonstrate that the PCNA^S228I^ variant affects all PIP box-PCNA interactions tested. Importantly however, the binding of p21 to PCNA is only minimally perturbed by PCNA^S228I^. Thus the PARD mutation has differential effects on PCNA’s ability to bind to its partner proteins.

### 3.3 The IDCL structure of PCNA is perturbed by the S228I change

**Fig. 4:**
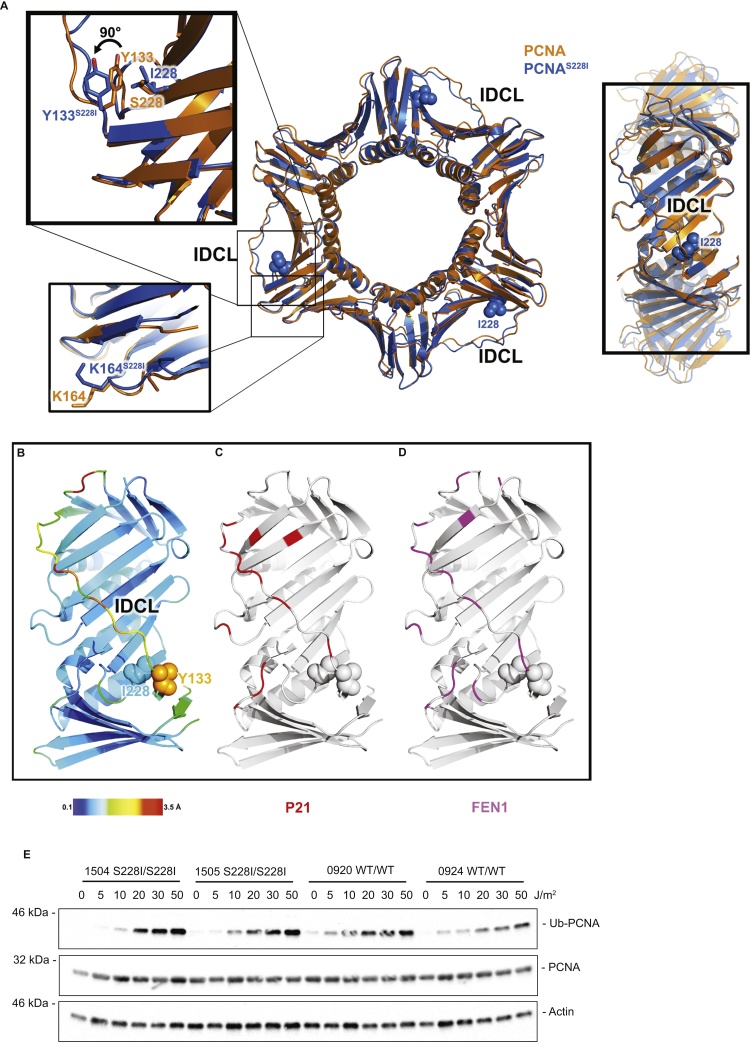
PCNA^S228I^ alters PCNA function via structural changes at the IDCL. A) Apo structure of PCNA^S228I^ (5MOM) shown in blue overlayed on apo PCNA^WT^[43] structure in orange. The mutated I228 residues are shown as spheres. Top left inset zoomed area shows the rotation of Tyr133, propagating along the IDCL. Bottom left inset zoom shows K164 and surrounding structural environment unchanged. Right panel shows the rotated view of the IDCL region highlighted in B, C, D below. B) Magnified view of the IDCL region of a single monomer of PCNA^S228I^ colour coded according to the heat map of the rmsd between PCNA^S228I^ (5MOM) and PCNA^WT^ (1VYM). C) As B, but with residues found at the interface between PCNA^WT^ and the p21 pip box (based on 4RJF) coloured in red. D) As C, but for the Fen1 PIP box (based on 4RJF), coloured in magenta. E) PARD affected and control lymphoblasts were treated with the indicated doses of UVC and harvested after 7 h. Western blot shows induction of mono-ubiquitinated PCNA^WT^ and PCNA^S228I^.

In none of the previously reported structures of PCNA bound to client proteins does the interaction interface involve serine 228, the residue which is altered in PARD. These include structures of PCNA^WT^ bound to p21 (1AXC and 4RJF), PolD3^p66^ (1U76), Fen1 (1U7B and 1UL1), Polι (2ZVM), Polη (2ZVK), Polκ (2ZVL), RNaseH2 B (3P87), p15^PAF^ (4D2G), DVC1 (5IY4) and TRAIP (4ZDT) [7], [15], [49], [50], [51], [52], [53], [54], [55]. To explain how the disease causing mutation can have such dramatic effects on PCNA function via altered client binding, we obtained a crystal structure of PCNA^S228I^ at 2.27 Å resolution (5MOM, Fig. 4A–D, Table 1). The overall structure of PCNA is retained by PCNA^S228I^, it is a recognisable toroidal trimer, with pseudo-6 fold symmetry and a central hole through which double-stranded DNA will fit (Fig. 4A). There is no evidence of trimer destabilisation, as the monomer–monomer interface appears unperturbed when compared to published structures of PCNA^WT^. This is consistent with our previous work, as we did not detect differences in trimer formation or stability [36]. Furthermore, we do not observe reduced levels of PCNA in cells from PARD affected individuals (see Fig. 4E and Ref. [36]) and in the presence of cycloheximide PCNA^S228I^ appears to be as stable as the WT protein (data not shown).

The structure is of sufficient resolution that the electron density of the isoleucine substitution can be identified (Fig. 4A). Comparison of this and the adjacent residues with the reported structure of PCNA^WT^ containing serine at this position (1VYM) provides a clear mechanism for the defective interactions resulting from the PCNA^S228I^ change. The larger isoleucine side chain has a steric clash with Tyr133 in the standard position, forcing a 90° rotation of the sidechain of Tyr133, which in turn repositions a significant proportion of the IDCL (amino acids Gly127 to Asp120). The S228I change has very little effect on the overall structure and whilst the total rmsd between our S228I structure and wild type structure is 0.6 Å, the local rmsd averaged over the three IDCL regions (residues 120–130) is 1.9 Å (Fig. 4B). Many of the residues of PCNA that are most affected by the S228I change coincide with those IDCL residues sitting at the interface between PCNA and PIP box containing proteins, such as p21 (Fig. 4C) and Fen1 (Fig. 4D). Recently a highly similar structure of PCNA^S228I^ was reported [47] and our data confirm those results. It thus appears that this dramatic structural reorganisation of the IDCL, resulting from the single amino acid change in the PCNA^S228I^ protein is likely to prevent this binding surface from operating correctly. This provides a structural explanation for the reduced binding of multiple PIP-boxes to PCNA^S228I.^ However, it does not explain why p21 is less affected by this amino acid substitution, which we explore later. We next turned our attention to post translational modifications and whether these were affected by PCNA^S228I^.

### 3.4 PCNA^S228I^ is ubiquitinated after DNA damage

PCNA can be mono-and polyubiquitinated and SUMOylated [56], [57], [58]. The major post-translation modification of PCNA in human cells is mono-ubiquitination at Lys164, which is generated in response to UV-damage by Rad6/Rad18 [59], [60], [61]. This modification is crucial for translesion synthesis and the tolerance of UV-induced DNA damage in replicating cells [62]. The position and surrounding structural context of Lys164 is unaltered by the PCNA^S228I^ substitution, (Fig. 4A) suggesting that PCNA^S228I^ likely remains competent for ubiquitination. However, there are multiple PCNA-PIP box interactions that occur in the vicinity of DNA replication forks that can influence PCNA ubiquitination and thus the PCNA^S228I^ mutation could indirectly affect PCNA ubiquitination events. We therefore assessed the formation of UV-induced ubiquitinated PCNA in cells from affected individuals and controls. We observed a robust induction of ubiquitinated PCNA (for both WT and S228I) across a range of UVC doses (Fig. 4E) and recovery times (see Fig. 6) implying that the ability to ubiquitinate PCNA is unaffected in PARD cells, and cellular effects do not result from a defect in this pathway.

### 3.5 PCNA^S228I^ does not remove a phosphorylation site

Given that the PCNA^S228I^ alteration removes a serine from PCNA which could potentially be phosphorylated we investigated whether this site is a target for phosphorylation in PCNA^WT^. Using a stably expressing Streptag-PCNA^WT^ cell line we isolated PCNA trimers, which will contain both tagged PCNA^WT^ and untagged endogenous WT monomers, and performed MS analysis to detect post translational modifications. We found a number of modifications including phosphorylation of Thr185 but no peptide containing phosphorylation of S228 was observed (Supplementary Figs. 4 and 5). Furthermore S228 is not reported modified in the published databases such as Uniprot, PhosphoSitePlus, Proteomics DB, or PHOSIDA. As we have not found any evidence of S228 being phosphorylated in our experiments or the public domain, it is unlikely that the observed phenotypes of PCNA^S228I^ are associated with the elimination of a putative phosphorylation site. Together, these data suggest that it is the direct structural alteration of the IDCL region, rather than loss of a phosphorylation site that is the molecular cause of the cellular defects underlying PARD. We thus went on to investigate the effect of the PCNA^S228I^ mutation on PCNA-client protein binding in more detail, and in particular the differences between Fen1 and p21.

### 3.6 The residues of the p21 PIP box confer PCNA^S228I^ resistant binding

**Fig. 5:**
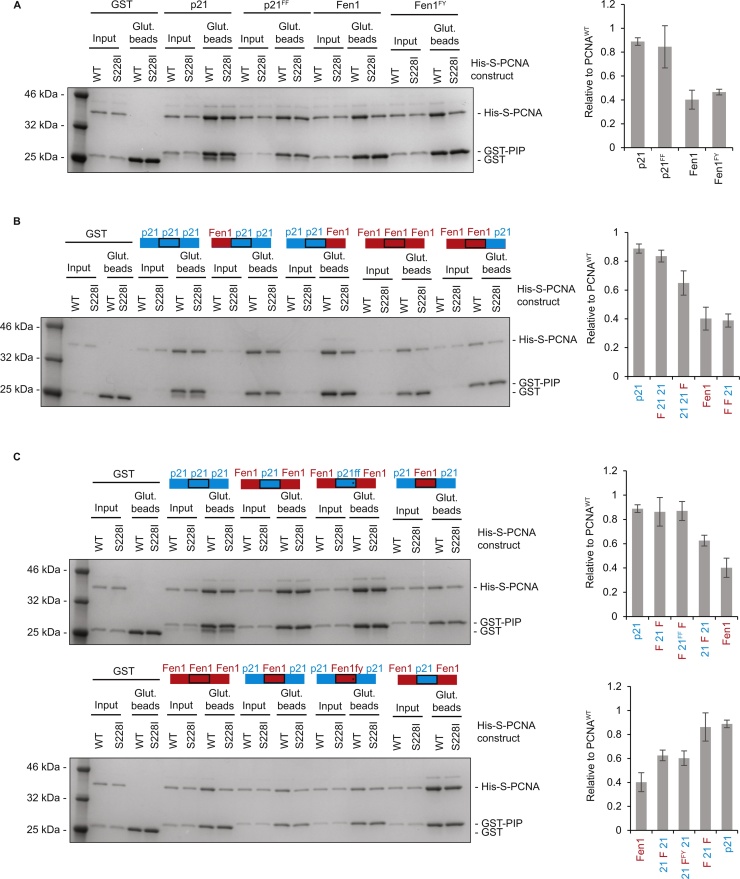
The PIP-box motif is the major contributor which enables p21 binding to PCNA^S228I^. A) GST-PIP pull down of His-S-PCNA^WT^ or His-S-PCNA^S228I^ using wild type p21 and Fen1, and constructs with mutated PIP position 8 residues. Figure shows Coomassie stained gel of representative pull down. Amount of ‘input’ and ‘Glut. beads’ as Fig. 2. Molecular weight markers are indicated. Histogram (right) shows pulldown of PCNA^S228I^ relative to PCNA^WT^ for indicated PIP constructs. p21 and Fen1 (n = 8), p21^FF^ (n = 4), Fen1^FY^ (n = 2). Error bars are standard deviation. B) As A) except for single domain swaps. For histogram: all n = 3 except p21 and Fen1 n = 8. C) As A) except for double domains swaps. For histograms: all n = 3 except p21 and Fen1 n = 8.

The interaction between p21 and PCNA is well characterised. In the crystal structure of human PCNA with a 22 amino acid peptide derived from p21 (1AXC), the PIP box and flanking regions form an expanded binding interface (>17 residues). p21-PCNA binding is also reported to have the highest affinity of any of the PCNA interactions, in large part due to the interactions made by Tyr151 of the p21 PIP box [49]. To identify the molecular determinants that allow the p21 PIP box to retain binding to PCNA^S228I^ we performed GST-PIP peptide pull downs using mutated versions of p21, and domain swaps between the Fen1 and p21 PIP boxes and flanking regions implicated in PCNA binding (Fig. 5, Supplementary Fig. 6). A GST-p21 PIP construct with Tyr151 mutated to phenylalanine (p21^FF^) retained its ability to bind PCNA^S228I^ to the same extent as p21 ^WT^ (Fig. 5A). In addition, a Fen1 PIP box construct in which the Phe344 is changed to tyrosine (Fen1^FY^) did not restore the ability of the Fen1-PIP to bind PCNA^S228I^ (Fig. 5A). This suggests that the presence of a tyrosine at position 8 of the PIP box does not in itself enable p21 to retain binding to PCNA^S228I^.

The high affinity binding of p21 to PCNA has also been ascribed to residues C-terminal to the PIP box in p21 which form a β-sheet with IDCL residues Met119 to Gly127 [15]. Fen1 in comparison is unable to form this extensive β-sheet [50]. N-terminal PIP flanking residues in p21 (specifically Ile255 to Ser261) also contact PCNA through ionic interactions, but these are poorly ordered so cannot be resolved to specific residues [15]. To examine the impact of these regions on mediating interactions between p21 and PCNA^S228I^ we constructed hybrid PIP-box GST fusion proteins comprising domain swaps between p21 and Fen1 and assessed their PCNA binding capabilities by GST pull down (Fig. 5B). Exchanging the p21 N-terminal flank or the C-terminal flank for that of Fen1 both slightly decreased the ability of p21-PIP to bind PCNA^S228I^ relative to PCNA^WT^, but binding was still significantly better than for Fen1. Exchange of the p21 C-terminal flank caused the slightly larger of the two reductions but this region did not improve Fen1 binding to PCNA^S228I^ (F F 21, Fig. 5B). Therefore, although both p21-PIP flanking regions contribute to full binding of p21 to PCNA^S228I^, they are not sufficient for this ability. Even the exchange of both p21-PIP flanking regions alone or in combination with Tyr151Phe did not reduce the ability of p21-PIP to bind PCNA^S228I^ (F 21 F and F 21^FF^ F, Fig. 5C). Therefore, the actual PIP-box sequence itself of p21 is the major factor enabling binding to PCNA^S228I^. Indeed, it is only when we exchanged the p21-PIP box for that of Fen1 (retaining p21 flanking regions) that we significantly reduced relative binding of p21 to PCNA^S228I^ (21 F 21, Fig. 5C upper panel), and in parallel, when we exchanged the Fen1-PIP box for that of p21 that we were able to induce significant binding of Fen1 to PCNA^S228I^, comparable to that of p21 itself (F 21 F, Fig. 5C lower panel).Thus although there is a small contribution from p21-PIP flanking regions, the major determinant of the ability to bind PCNA^S228I^ lies within the 8 amino acid PIP box motif of p21.

### 3.7 PCNA^S228I^ has only a minor effect on the stability of Cdt1 and p21

In the last decade it has become clear that a unique class of PIP boxes, the PIP degrons, couple the binding of PCNA to protein degradation in order to ensure regulated progression through the cell cycle (reviewed in Ref. [63], [64]). PIP degron motifs are similar to canonical PIP-boxes but they specifically contain a threonine at position 5, aspartic acid at position 6 and a positively charged residue at position +4 (C-terminal) [48]. The targeted proteasomal degradation of PIP-degron containing client proteins following binding to PCNA is achieved through recruitment of the Cdt2^CRL4^ ubiquitin ligase complex (reviewed in Ref. [63], [64]), either after DNA damage or on entry into S-phase. Client proteins containing PIP degrons include p21, the pre-replication complex component Cdt1, and the histone methyltransferase Set8 [13], [65], [66], [67], [68], [69], [70], [71], [72], [73], [74], [75], [76]. PolD4^p12^, a component of the Polδ holoenzyme; Cdc6, a component of the pre-replication complex; and the translesion synthesis polymerase Polη are also suggested to contain PIP-degrons [77], [78], [79], [80], [81].

**Fig. 6:**
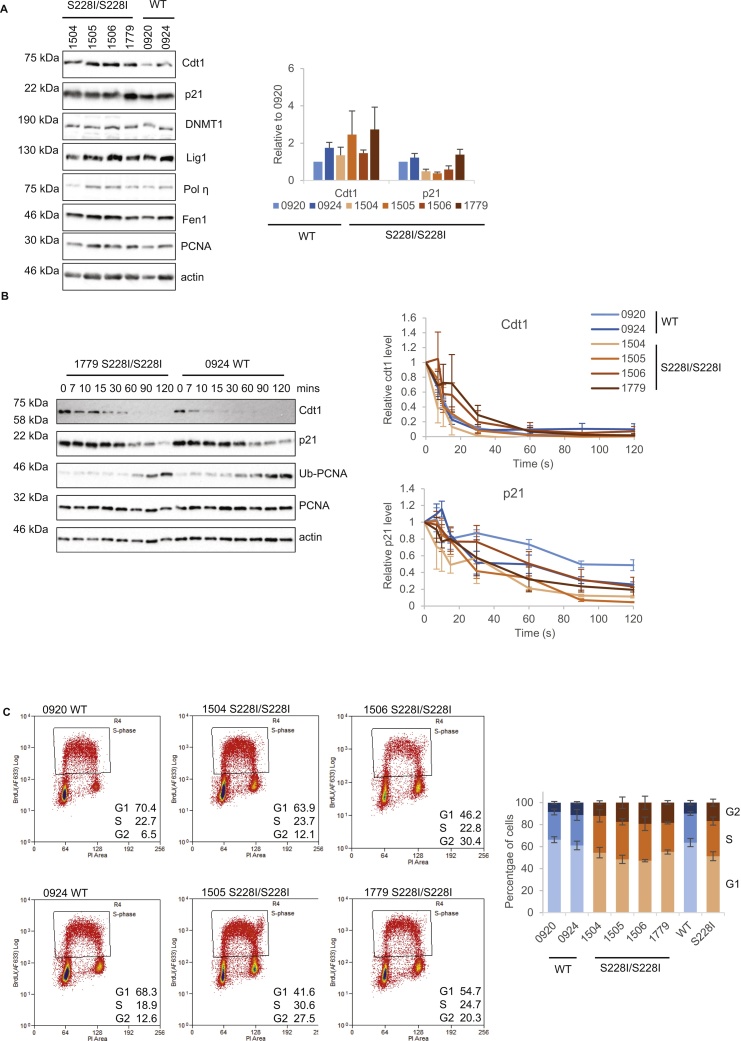
PCNA^S228I^ has subtle effects on cellular functions of PCNA. A) Example Western blots showing steady state levels of PCNA interacting proteins as indicated, also levels of PCNA and actin as a loading control. Histogram shows quantification of p21 and Cdt1 levels relative to actin, shown relative to 0920 levels. n = 8 (0920, 0924), n = 7 (1504, 1505), n = 6 (1506), n = 5 (1779). B) Example western blots showing degradation of Cdt1 and p21 at indicated times after 50 J/m^2^ 254 nm UV exposure. Also levels of PCNA and actin as loading controls and appearance of ubiquitinated-PCNA at later time points. The graphs show quantification of p21 and Cdt1 levels relative to actin, expressed as relative to time zero. n = 3 (1504, 1505, 1506, 1779), n = 5 (0920, 0924). C) Representative dot plots from 3 independent FACS analysis for indicated cell lines plotting DNA content (PI, x-axis) against BrdU (y-axis) to determine percentage in G1, S and G2 cell cycle phases. Graph (right) shows average cell cycle proportion for each cell line (error bars are SEM) and each genotype (WT and S228I, error bars are STDEV).

We have shown that the interaction between Cdt1 and PCNA is perturbed in vitro by the S228I mutation, while the p21 interaction is relatively unaffected (Fig. 2, Fig. 3). We thus hypothesised that Cdt1 might be less well targeted for appropriate degradation in cells carrying PCNA^S228I^ whereas p21 degradation might be unaffected or even increased due to reduced competition for PCNA binding. To assess this we firstly determined the steady state levels of these proteins in cells from PARD-affected individuals and controls. However, we do not find significant differences in the steady state levels of Cdt1 or p21 that are consistent across genotypes. We note that there is a small tendency for increased Cdt1 and for reduced p21 levels in PARD cell lines (Fig. 6A), however the biological significance of this is not yet clear. We did not observe any other steady state differences in any other PCNA client proteins tested (Fig. 6A).

The PCNA-mediated degradation of Cdt1 and p21 is induced after UVC treatment [65], [67]. Thus we analysed the decrease in the levels of these proteins after UVC irradiation of cells carrying PCNA^S228I^ and PCNA^WT^. In all cell lines Cdt1 was degraded more rapidly than p21 (Fig. 6B), as previously reported [82]. Remarkably considering the large differences in binding affinities from our in vitro work, we do not observe dramatic alterations in the rate of Cdt1 or p21 degradation that is consistent across genotype. (Fig. 6B). It is of note that multiple mechanisms control Cdt1 levels to prevent re-replication, including a pathway of Cdt1 degradation utilising the SCF^Skp2^ ubiquitin ligase complex [73]. It is therefore possible that we do not observe dramatically slower Cdt1 degradation in PCNA^S228I^ cells because redundant mechanisms operate to ensure the timely destruction of this crucial replication control factor. Alternatively, these data may suggest a strong robustness in (at least) some of the activities of PCNA if the residual binding of Cdt1 to PCNA^S228I^ is still sufficient to ensure timely degradation. This interesting scenario will be the focus of further work.

Lastly we show small but consistent differences in cell cycle phase distribution between PCNA^S228I^ and WT control lymphoblasts. Representative data are shown in Fig. 6C with summary graph from three experiments. PCNA^S228I^ lymphoblasts show a slight reduction in G1 and increase in both S and G2 populations (Fig. 6C). This might suggest some reduced ability to progress through S-phase but requires further investigation in a more tractable system. Interestingly, we did not observe such alterations in cell cycle phase distribution when using primary fibroblasts from affected individuals and controls [36]. We hypothesise that the low proliferation rate of these primary fibroblasts masked the S phase dependent effects that we now observe.

Here we have shown that the PCNA^S228I^ mutation has implications for PCNA-binding events that are not related to DNA repair. We have identified DNMT1, Cdt1, RNaseH2B, PolD3^p66^ and PolD4^p12^ as PIP box containing proteins that are affected in their ability to bind to PCNA^S228I^, and provided a structural explanation for the reduced binding capability of PCNA^S228I^. The p21 PIP box has an unusual property in that it is able to bind with relatively normal affinity to PCNA^S228I^ and we have demonstrated that this ability is derived from the PIP box itself. In spite of the dramatic effects on in vitro PCNA binding events caused by PCNA^S228I^ the cellular phenotypes of this mutation are only subtle. Nevertheless, cells from individuals affected by PARD are sensitive to the PCNA inhibiting compounds T3 and T2AA and have consistent differences in cell cycle phase distribution. These effects should be considered in attempts to explain the etiology of PARD.

## 4 Discussion

### 4.1 Competition between PIP-box containing proteins for PCNA binding

The interaction between PCNA and many of its client proteins is extremely well characterised at the structural level with more than 70 crystal structures, from more than 10 species, deposited in the PDB. However, the sheer number of proteins reported to be able to interact with PCNA, the trimeric nature of the PCNA ring, and the dynamic and competitive nature of the binding events mean that there is a much more limited in vivo understanding of the precise role that PCNA plays in dynamically regulating essential activities during replication and repair. The identification of the PCNA^S228I^ mutation in a human population has therefore given us a unique opportunity to investigate the in vivo role of PCNA in human cellular activities. The S228I alteration causes dramatic reductions in the binding abilities of most client proteins in vitro and yet the cellular phenotypes appear to remain relatively subtle. This suggests that, in the competitive nuclear environment, even reduced binding affinities are sufficient to drive essential interactions with PCNA. Of course, if all PCNA-PIP interactions are equally perturbed by the S228I mutation then, in a competitive environment with sufficiently high concentrations of client proteins, there might be relatively little alteration to the relative final binding profile: the reduced affinity interactions would all occur in the circumstances of reduced competition. This might account for the ability of the PCNA^S228I^ cells to survive in the face of an apparently dysfunctional PCNA. We have already commented that the structural organisation of replication factories, providing high local concentrations of PCNA, can also go some way towards accounting for the relatively specific effect that the S228I mutation has on repair, as opposed to replicative functions [83].

However, it is important to note that the S228I mutation does not affect all PCNA-PIP box interactions equally. Notably the p21 PIP box seems relatively blind to the effect of this amino acid change. p21 is reported to have one of the highest affinities for PCNA, suggesting that the p21-PCNA interaction has evolved to outcompete alternative PIP-mediated protein binding events. In PCNA^S228I^ cells the reduced affinity for other PIP-box proteins would mean that p21 is even more likely to gain control of the PCNA binding interface. Ectopic expression of high affinity, non-degradable PIP-box-containing fusion proteins or peptides perturbs the equilibrium of PCNA interactions and has profound effects on cell proliferation [84], [85]. Of course, p21 contains a PIP degron and normally the PCNA associated p21 is efficiently degraded, clearing the way for subsequent association of the next client protein. We have shown that p21 degradation is operational in PCNA^S228I^ cells, and this should help to mitigate the consequences that would otherwise arise from a hypercompetitive PCNA binder.

### 4.2 p21 retains binding to PCNA^S228I^

p21 makes extensive contacts with PCNA using regions flanking the PIP-box, however our results from Fen1 and p21 domain swap experiments show that the retained ability of p21 to bind to PCNA^S228I^ is mostly derived from the PIP box itself. It is as yet unclear whether it is particular residues at specific positions within the PIP-box or the combination which is important for this ability. p21 is a PIP-degron, however the TD that is part of the PIP-degron motif in p21 cannot be responsible since it is also found in Cdt1, the binding of which is affected by the S228I mutation. Comparing the PIP box sequences of the proteins that we have tested, it is only the methionine at position 4 which is unique in p21, and thus which represents a good candidate for mediating this binding activity. In the crystal structure of the p21 PIP with PCNA, this methionine (Met147) is not reported to form specific interactions with PCNA [15], but it is possible that a methionine at this position, perhaps in combination with the other p21 specific residues, generates a PIP box with the specific attributes that enable binding to the abnormal IDCL of PCNA^S228I^.

### 4.3 Different classes of PIP-boxes

In our hands GST-PIP peptides from two reported PCNA-interacting proteins (Cdc6 and Cdt2), did not interact with PCNA in a GST-pulldown assay, even when assayed using the same buffers as previously published experiments. These proteins may form a separate class of PCNA binders that either require additional sequences not included in our GST-PIP constructs (-12, +9 residues) or binding levels may be below the level of detection in this assay but might be revealed in other less stringent assays. Alternatively, these proteins may require PCNA bound to chromatin for successful binding, or post-translational modifications, for example polη binds preferentially to mono-ubiquitinated PCNA [61].

### 4.4 PCNA-associated repair disorder

The data presented here, and in our previous work, show that the dramatic deficiencies in client protein binding caused by the S228I mutation in vitro do not result in comparably dramatic cellular phenotypes. This indicates that PCNA functions in cells are impressively robust, able to operate even if binding affinities are dramatically reduced. However, there are some important consequences of this mutation because PARD has severe clinical phenotypes. Cells derived from individuals affected by PARD are more sensitive to UV irradiation, and show reduced unscheduled DNA synthesis and recovery of RNA synthesis after UV [36]. These defects in NER are likely linked to the disrupted binding of PCNA^S228I^ to the key NER factor XPG, as well as possibly Lig1 and Fen1 which are also implicated in excision repair processes. The fact that cells with PCNA^S228I^ show increased sensitivity to PCNA inhibition, and an altered cell cycle profile also implies that the reduced affinity of PCNA^S228I^ for its clients can manifest as consequences in undamaged cells. It certainly seems likely that, occasionally during cellular replication, there will be a relative imbalance in PCNA client availability, which will then be particularly problematic when PCNA’s binding ability is compromised by S228I. In a similar fashion, the subtle effects seen on PIP degron proteins may result in occasional errors in controlling entry into S phase. Such rare stochastic failures in PCNA function may not have dramatic consequences for cells in culture, but a consideration of such events will certainly be important as we try to understand the development and clinical manifestations of the PCNA associated repair disorder.

## Funding

This work was supported by the Medical Research Council [MR/L006812/1 to CMG, RHCW, ELB, AHC]; the Wellcome Trust [studentship 099667/Z/12/Z to AJB, 090532/Z/09/Z to CMG]; the Nuffield Department of Medicine, Oxford, UK [studentship to LW]; and the Kennedy Trust Fund [RF]. Funding for open access charge: [Wellcome Trust].

## Figures and Tables

**Table 1:** Data collection and refinement statistics (molecular replacement). Numbers in parentheses refer to the highest resolution shell.

	hPCNA^S228I^ (5MOM)
Data collection	
Space group	P 4_3_ 2_1_ 2
Cell dimensions	
*a*, *b*, *c* (Å)	162.95, 162.95, 140.40
α, β, γ (°)	90.00, 90.00, 90.00
Wavelength	0.9762
Resolution (Å)	89.07–2.27 (2.33–2.27)
*R*_sym_ or *R*_merge_	0.019 (0.122)
*I/σI*	17.7 (1.5)
Completeness (%)	100.0 (100.0)
Redundancy	13.4 (13.2)

Refinement	
Resolution (Å)	89.07–2.27
No. reflections	87374
*R*_work_/*R*_free_	0.1987/0.2160
No Atoms	
All (non-Hydrogen)	5731
Protein	5579
Solvent	152
B-factors	
Protein	62.5
R.m.s. deviations	
Bond lengths (Å)	0.008
Bond angles (°)	1.126
Ramachandran Statistics	
Favored (%)	97.42
Permitted (%)	2.58
Outliers (%)	0
