# Supplementary material for: PCNA dependent cellular activities tolerate dramatic perturbations in PCNA client interactions
Source: DNA Repair (Amst). 2017 Feb;50:22–35. doi: 10.1016/j.dnarep.2016.12.003 (PMC5264654; doi:10.1016/j.dnarep.2016.12.003)
Supplement: Supplementary file 1 [file mmc1.pdf]

Supplementary Figure legends

Supplementary Figure 1. Testing of additional PIP box proteins and conditions. A) Schematic showing GST-PIP peptide constructs. GST represented by rectangle, PIP box sequences shown in red. Numbers relate to amino acid position in full protein. B) GST-PIP pull down of His-S-PCNA^{WT} or PCNA^{S228I}. Figure shows Coomassie stained gel of representative pull down using 100 µg GST-PIP constructs. Amount of 'input' and ' Glut. beads' as Fig 2. C) GST-PIP pulldown of GST-Cdt2-PIP peptide as (1). D) Figure shows Coomassie stained gel of GST or GST-Cdt1 PIP pull down of His-S-PCNA^{S228I}. Samples are as Fig 2 but with a higher percentage of bead sample loaded. Amount of 'input' loaded is equivalent to 1%,' Glut. beads' (Glutathione sepharose 4B beads) is equivalent to 65%. Molecular weight markers are indicated. E) Figure shows Coomassie stained gel of GST-PIP pulldown of His-S-PCNA^{WT} or His-S-PCNA^{S228I} performed in the presence of 20 µg of HpaII digested plasmid DNA.

Supplementary Figure 2. Raw SPR curves used to calculate affinity curves shown in Fig 3. Graphs show Response Units (RU) recorded from SPR experiments for the indicated GST-PIP peptide coupled lanes (A-G) and for concentration ranges of either His-S-PCNA^{WT} or His-S-PCNA^{S228I}. Colours shown on graphs correspond to PCNA concentrations indicated in the key (bottom). Some spurious spikes of machine fluctuation origin were removed from the data for clarity, however only within timepoints between 50-220 secs, ie nothing was removed at the key point of equilibrium calculation at the end of association phase. Note also that these spurious spikes were not removed from data before affinity curve calculation, but purely for ease of viewing the raw data as shown here.

Supplementary Figure 3. SPR in reverse orientation also shows reduction in PIP binding to PCNA^{S228I}. A) Affinity curves (i) and corresponding raw single cycle kinetics SPR data (ii) are shown for reverse SPR, where 3-tag PCNA^{WT} (black) or PCNA^{S228I} (red) was bound to SA chip lanes and either GST or GST-p21-PIP peptide used as the analyte. Buffer only shown in pink and grey on ii. B) As A) but for GST-Cdt1-PIP peptide. Calculated K_Ds are indicated.

Supplementary Figure 4. Mass spectrometry analysis of PCNA^{WT} shows that the S228I mutation does not cause loss of a phosphorylation site. A) Coomassie stained gel of Strep-pulldown of Streptag-V5-PCNA and endogenous PCNA – bands indicated by box. Both bands were excised and sent for Mass Spectrometry analysis. B) Anti-PCNA western blot shows pulldown of Strep-V5-PCNA and endogenous PCNA specifically from Strep-V5-PCNA expressing cells. C) MS/MS spectrum of a peptide covering S228 demonstrating good detectability and fragmentation of tryptic peptide. The y11 fragment containing S228 generates a well detected fragment ion. D) Multiple peptides covering S228 were detected. However, we did not observe potential baseline phosphorylation of S228.

Supplementary Figure 5. Full Mass Spec results Following LC-MS/MS analysis we were able to detect PCNA with a sequence coverage of 83% with only partial coverage of the N-terminus. S228 is covered by a series of tryptic peptides, sometimes with C- and N-terminal extensions. We detected phosphorylation on T185 but not S228.

Supplementary Figure 6. Schematic showing GST-PIP peptide constructs used for p21 and Fen1 domain swap experiments. GST represented by rectangle, PIP box sequences underlined, p21 sequences shown in blue, Fen1 sequences shown in red. Boxes highlight PIP positions 7 and 8. Numbers relate to amino acid position in full length protein.

Supplementary References

1. Kim, D.H., Budhavarapu, V.N., Herrera, C.R., Nam, H.W., Kim, Y.S. and Yew, P.R. (2010) The CRL4Cdt2 ubiquitin ligase mediates the proteolysis of cyclin-dependent kinase inhibitor Xic1 through a direct association with PCNA. *Molecular and cellular biology*, **30**, 4120-4133.

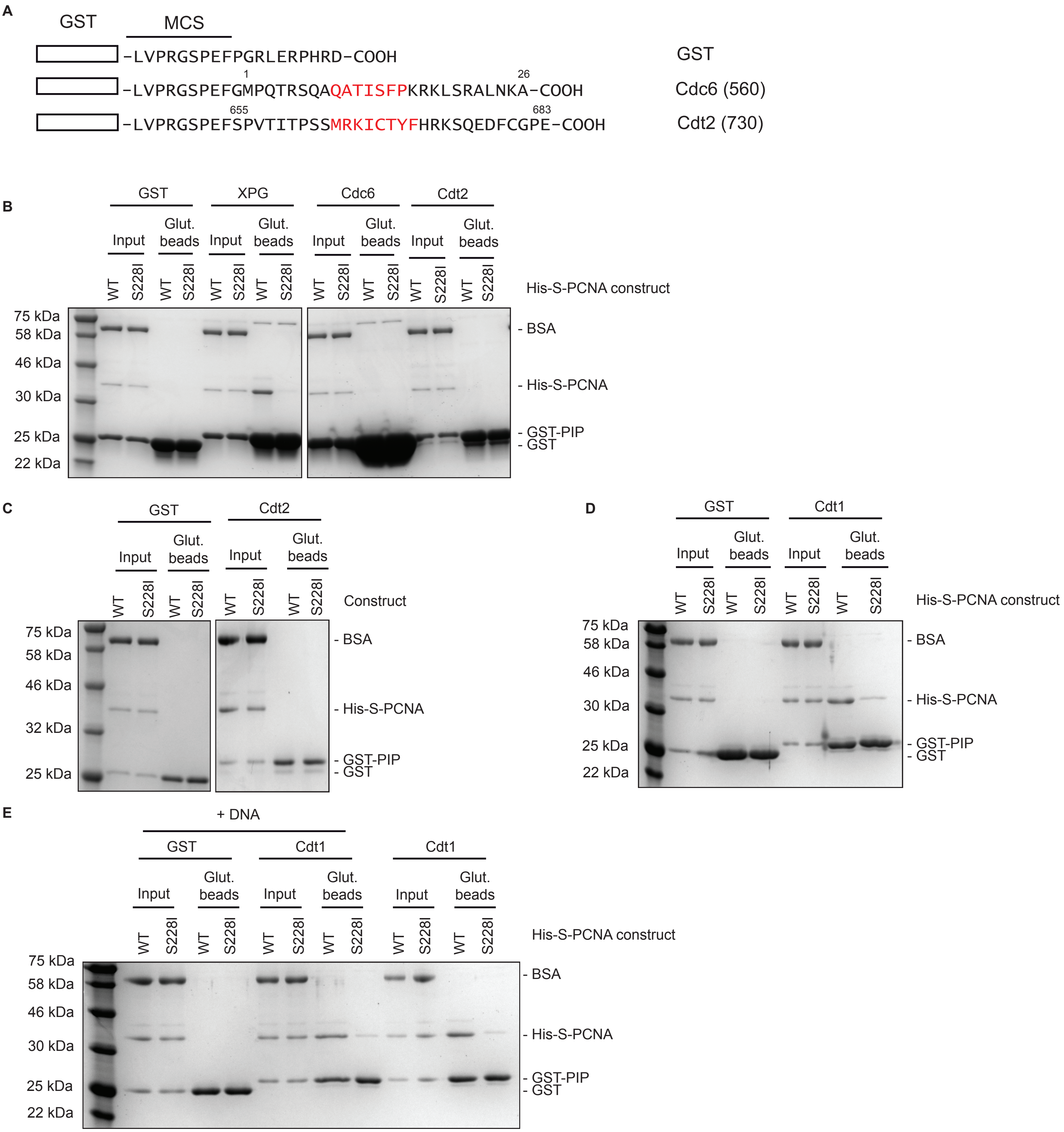

Supplementary Figure 1

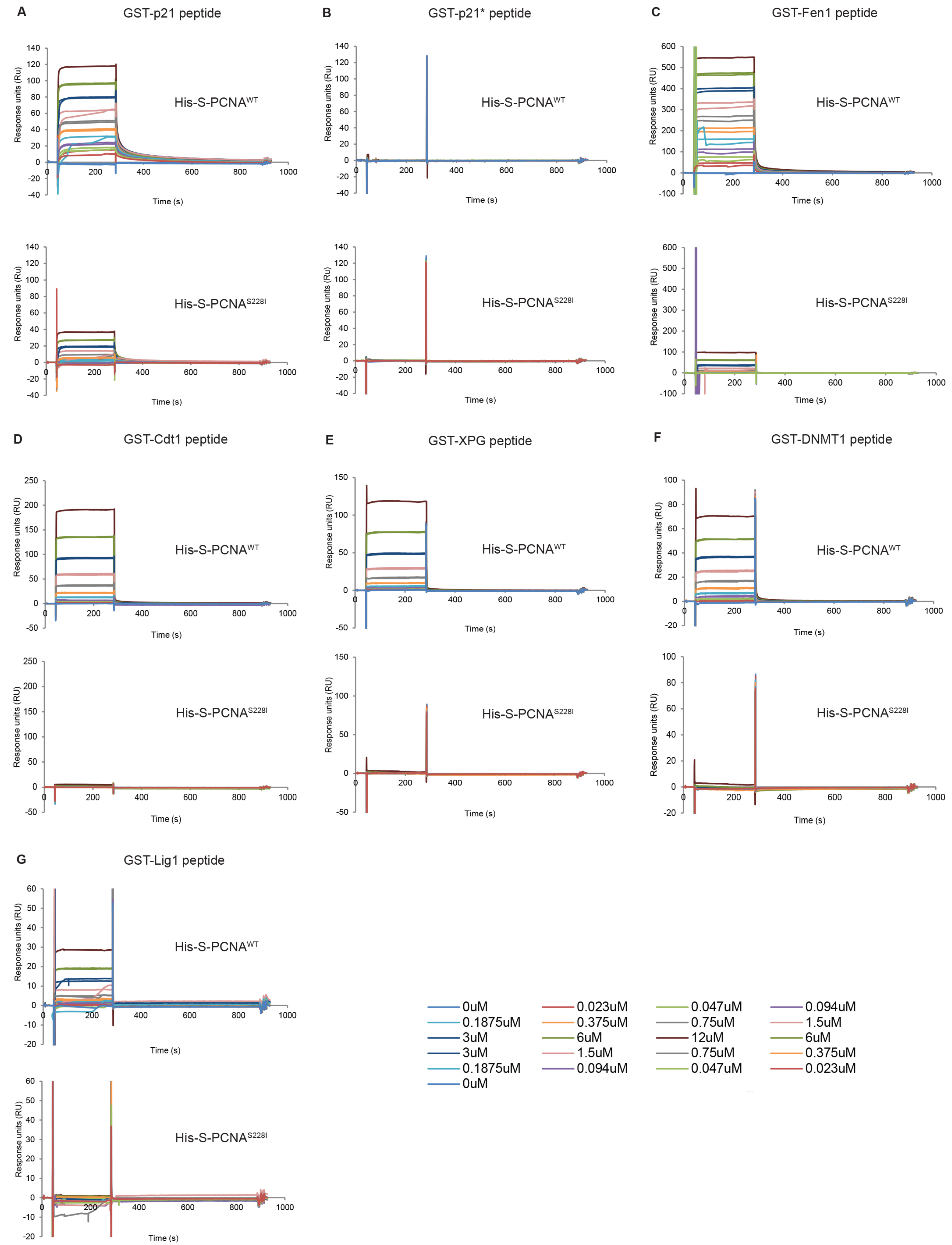

Supplementary Figure 2

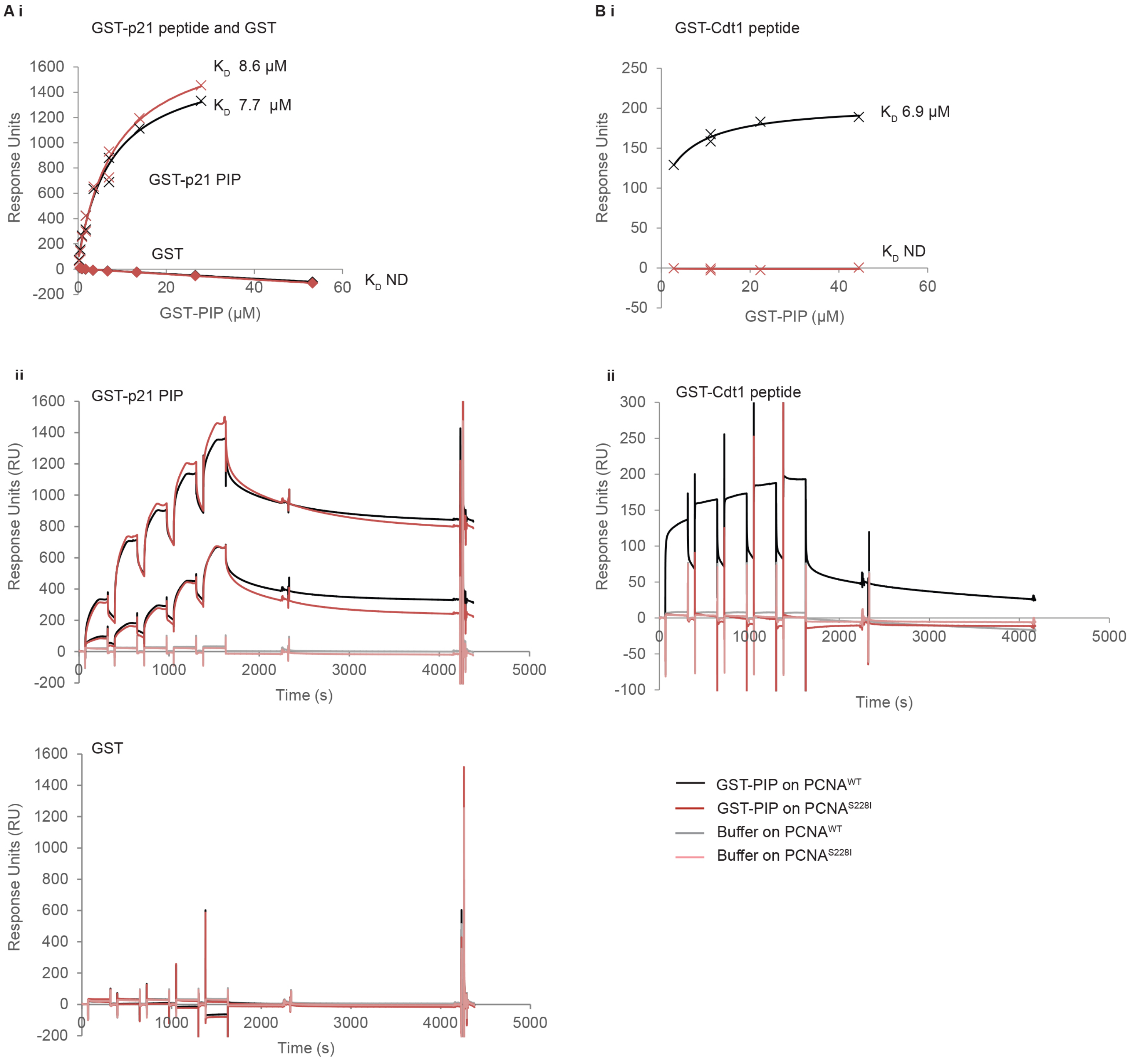

Supplementary Figure 3

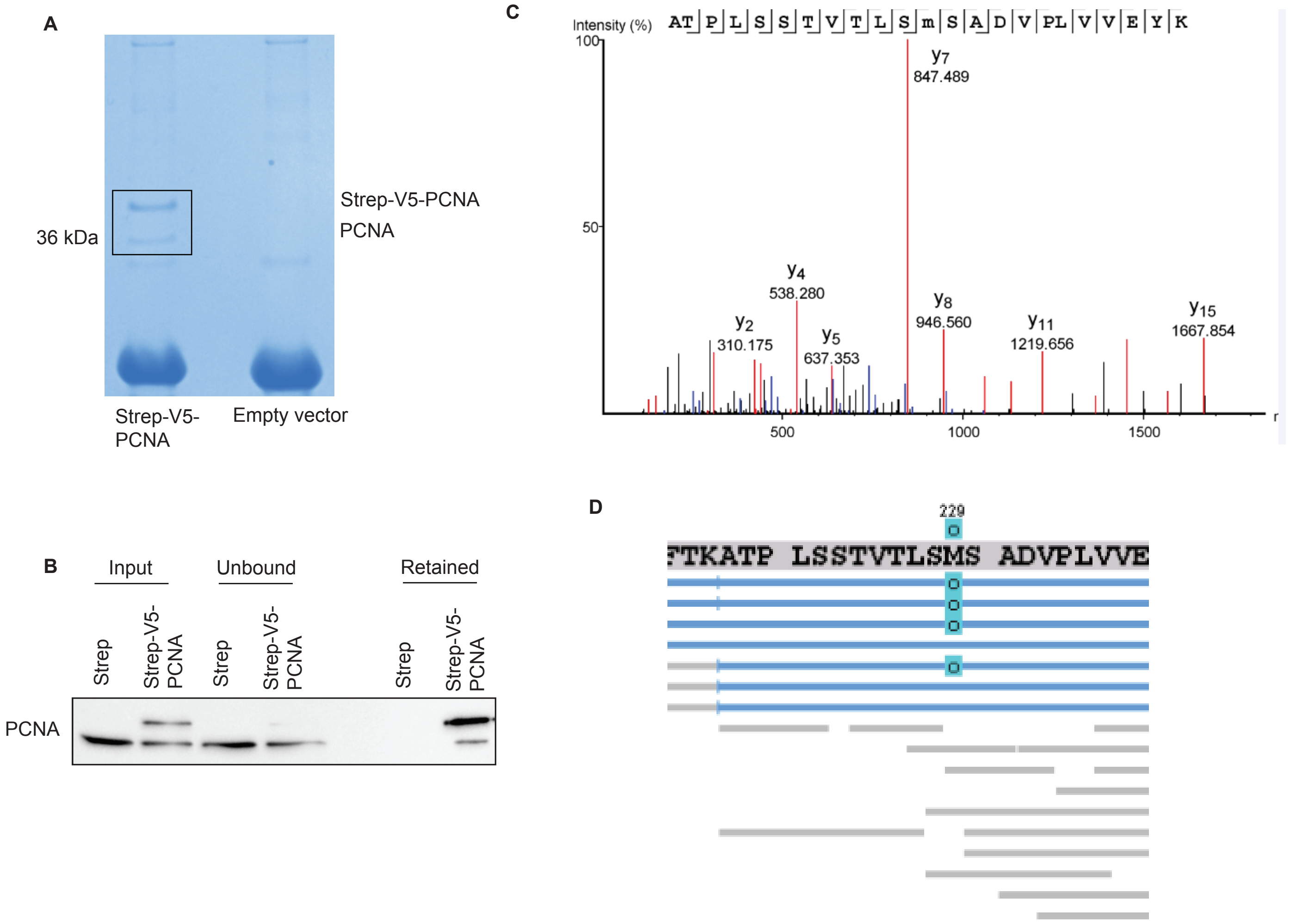

Supplementary Figure 4

Coverage 83%

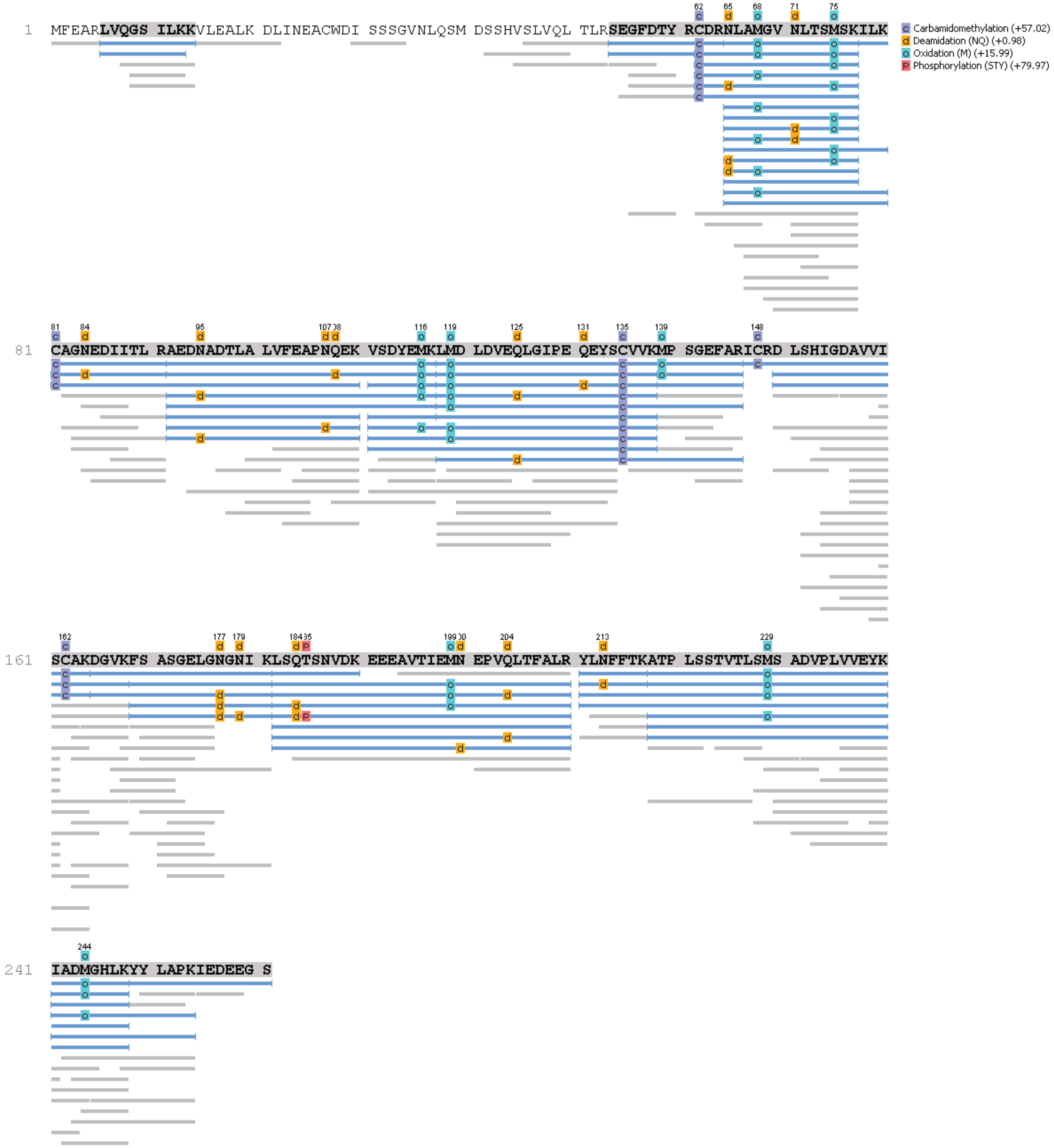

Supplementary Figure 5

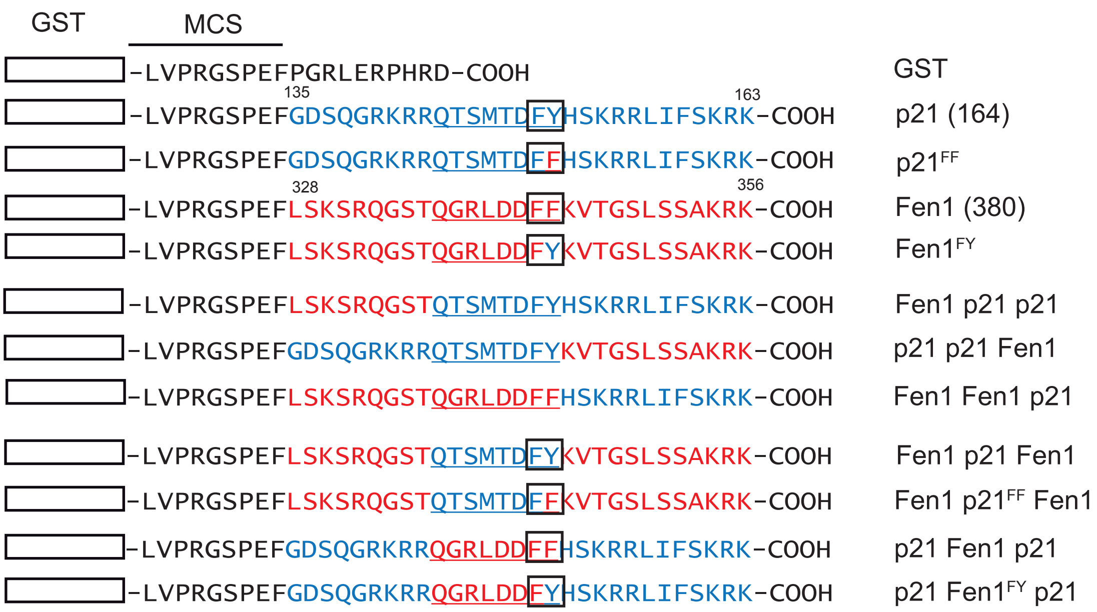

Supplementary Figure 6
